# Mammal-related *Cryptosporidium* infections in endemic reptiles of New Zealand

**DOI:** 10.1007/s00436-023-07824-4

**Published:** 2023-03-24

**Authors:** Juan C. Garcia-R, Anthony B. Pita, Niluka Velathanthiri, An Pas, David T. S. Hayman

**Affiliations:** 1 Molecular Epidemiology and Public Health Laboratory, Hopkirk Research Institute, Massey University, Private Bag 11–222, Palmerston North, New Zealand; 2 Auckland Zoo, Motions Rd, 1022 Auckland, New Zealand

**Keywords:** *Cryptosporidium hominis*, *Cryptosporidium parvum*, *Cryptosporidium serpentis*, Gecko, Skink, Tuatara, Zooanthroponosis

## Abstract

New Zealand’s endemic reptile fauna is highly threatened and pathogens causing infectious diseases may be a significant risk to already endangered species. Here, we investigate *Cryptosporidium* infection in captive endemic New Zealand reptiles. We found two mammal-related *Cryptosporidium* species (*C. hominis* and *C. parvum*) and six subtypes from three gp60 families (Ib, Ig and IIa) in 12 individuals of captive endemic Tuatara, Otago and Grand skinks, and Jewelled and Rough geckos. *Cryptosporidium serpentis* was identified in two Jewelled geckos using 18S. In New Zealand, *C. hominis* and *C. parvum* are associated with infections in humans and introduced domestic animals but have also been recently found in wildlife. Our finding of *Cryptosporidium* infection in endemic reptiles can help inform strategies to monitor the conservation of species and manage potential introductions of pathogens to *in-situ* and *ex-situ* populations.

**Supplementary Information:**

The online version contains supplementary material available at 10.1007/s00436-023-07824-4.

## Introduction

New Zealand has a diverse endemic terrestrial reptile fauna consisting of about 124 described species of lizards (geckos and skinks) and the Tuatara (Chapple et al. 2009; Hay et al. 2010; Hitchmough et al. 2021; Nielsen et al. 2011; O’Neill et al. 2008; Tingley et al. 2013; Towns et al. 2001). A significant majority of New Zealand’s reptile taxa are threatened with extinction (Hitchmough et al. 2010). Introduced mammal predators are the major driver of species declines and endangerment (Doherty et al. 2016; Tingley et al. 2013). However, there are other factors, including diseases, that can contribute to increasing the risk of extinction.

Gastrointestinal tract infections by protozoans can cause diarrhea, emaciation, anorexia, weight loss, and even death in some reptiles (Alley and Gartrell 2019; Gartrell 2016; Gartrell and Hare 2005; Scullion and Scullion 2009; Terrell et al. 2003). Cryptosporidiosis, for instance, can be chronic and sometimes lethal. Chronic cases show regurgitation, anorexia, and weight loss (Fayer et al. 1997; Fayer and Xiao 2008; Koudela and Modrý 1998). Cryptosporidiosis is caused by *Cryptosporidium* species, protozoal parasites that were first confirmed to infect reptiles in the 1970s (Brownstein et al. 1977) and currently recognized as a cause of gastrointestinal disease in a wide range of reptiles (Kváč et al. 2014; O'Donoghue 1995; Upton et al. 1989; Xiao et al. 2004).

The modes of transmission of *Cryptosporidium* in reptiles are the faecal-oral route including via direct contact between animals or through contact with contaminated objects (Graczyk et al. 1997; Xiao et al. 2004). The two most common species infecting reptiles are *C. varanii* (syn. *C. saurophilum*) (Pavlasek and Ryan 2008) and *C. serpentis*, both found in snakes and lizards (Fayer et al. 2000; Morgan et al. 1999; Ryan et al. 2021b; Xiao et al. 2004). Reptiles infected with *C. serpentis* may show symptoms of mild to severe gastritis with frequent regurgitation, particularly after feeding, while *C. varanii* causes enteritis and diarrhea. Other species, *C. ducismarci,* and *C. testudines* have been identified causing intestinal disease in tortoises (Ježková et al. 2016; Traversa 2010). A few other *Cryptosporidium* species and undescribed genotypes (e.g., mouse and tortoise genotypes) reported in reptiles were likely ingested through infected prey (Alves et al. 2005; Pedraza-Díaz et al. 2009; Richter et al. 2011; Rinaldi et al. 2012; Traversa et al. 2008; Xiao et al. 2004), including species associated with mammalian hosts, such as *C. parvum, C. tyzzeri,* and *C. muris* (Zahedi et al. 2016b).

The identification of *Cryptosporidium* species, and especially species associated with mammal infections, in endemic New Zealand reptiles is unknown. Here, we carry out a molecular epidemiological investigation to identify the species and subtypes infecting captive endemic reptiles of New Zealand.

## Methods

### Sampling

**Fig. 1:**
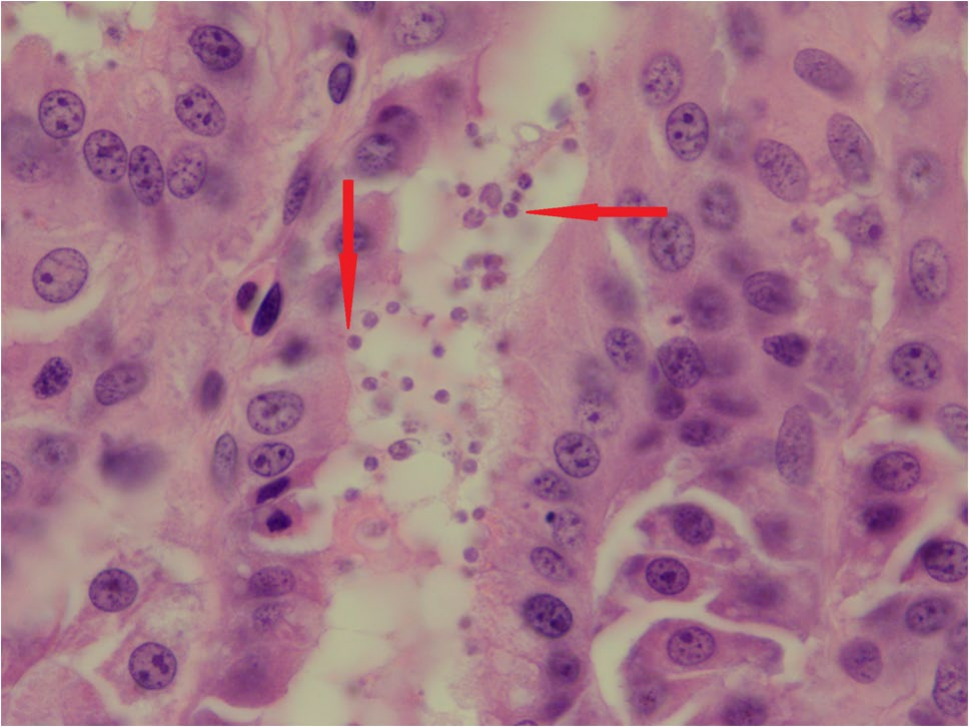
Optical images of the H&E stained tissue section diagnosed by pathology from the Jewelled gecko (*Naultinus gemmeus*) Lab ID 16,912. The arrows indicate oocyst of *C. serpentis.* Photo by courtesy of Cathy Harvey (Gribbles Veterinary NZ)

Between November 2018 and July 2021, we received 22 samples (faeces, intestinal tissue, and gastric or cloacal washes) from the Auckland Zoo (n = 17), Wellington Zoo (n = 2), the Wildbase at Massey University (n = 2) and Invercargill City Council (n = 1) from five New Zealand endemic reptile species including Otago skink (n = 9), Grand skink (n = 5), Jewelled gecko (n = 4), Tuatara (n = 3) and Rough gecko (n = 1) (Table S1). Samples were sent to the ^*m*^EpiLab at Hopkirk Research Institute (Massey University) for DNA extraction, PCR amplification, and sequencing. Histology diagnosis by a veterinary pathology laboratory (Gribbles Veterinary, Auckland) in tissues collected from only two clinically ill and dead Jewelled geckos in the Auckland Zoo (Lab IDs 16,911 and 16,912) suggested that gastritis and/or stomach inflammation was likely caused by *Cryptosporidium* (Fig. 1). All other samples were taken from animals that had no clinical signs of disease.

### DNA extraction, PCR, and sequencing

DNA was extracted as previously described (Garcia-R et al. 2017, 2020b; Garcia-R and Hayman 2017) using the Isolate faecal DNA (Zymo) kit following the manufacturer’s instruction. DNA extractions from reptile samples were carried out separately from human samples that our laboratory receives regularly. DNA extraction required physical disruption of the oocyst using a beadbeater (Tissue Lyser II, Qiagen) at 30 Hz for 5 min. The species and subtype of the isolates were identified by nested PCRs of the gp60 and 18S using a combination of external and internal primers (Table S2) (Glaberman et al. 2002; Johnson et al. 1995; Learmonth et al. 2004; Waldron et al. 2009; Xiao et al. 2000; Xiao et al. 1999) and sequencing of the secondary PCR products in both directions on an ABI 3730XL automated DNA sequencer (Applied Biosystems). Positive (consisting of human-derived *C. parvum*) and negative (consisting of all reagents minus template, which was replaced by nuclease free water) controls were included in each PCR run. All our PCR positive and negative controls were positive and negative, respectively. Consensus sequences were assembled from forward and reverse reads and edited manually using Geneious v.10.1.3 (Kearse et al. 2012). The sequences derived were used to identify species and subtypes by aligning to sequence entries in nucleotide databases using the program BLAST (http://www.ncbi.nlm.nih.gov/blast/; last accessed October 25, 2022) and checked by their corresponding subtype by maximum % identity. The sequences of the partial gp60 and 18S genes were deposited in the GenBank database under accession numbers OP778244-OP778255 and OQ457495- OQ457496, respectively.

## Results

Sequence analysis of the gp60 gene identified *C. parvum* and *C. hominis* subtypes in 12 of the 22 samples (Table 1). The remaining ten samples were gp60 PCR negative. Most reptiles were found with both *Cryptosporidium* species. The sequences obtained from isolates in endemic captive reptiles were found with a > 99% identity and an *e*-value of 0.0 to sequence data reported in GenBank. The subtypes are common in human infections (IbA10G2, IgA17, and IIaA18G3R1), however, we found subtypes of *C. parvum* with shorter (IIaA17G3R1) or similar (21 short tandem repeat region) but uncommon (IIaA17G4R1) repeats of the serine-coding trinucleotide*.* Only two samples from Jewelled geckos were successfully amplified using 18S primers (Table 1). These samples were ~ 99% identical to *C. serpentis* (accession number AF093499)*.* One of these geckos was also *C. hominis* positive by gp60 PCR (Table 1). Three other samples were amplified using 18S primers, but sequencing results ruled them out as fungi (~ 99% identity to Tremellomycetes or Basidiomycota).

**Table 1:** *Cryptosporidium parvum* and *C. hominis* genotypes found in endemic New Zealand reptiles

Lab ID	Host	Scientific name	Species (gp60/18S)	gp60 subtype	Accession numbers (gp60/18S)
18,421	Tuatara	*Sphenodon punctatus*	*C. hominis*	IbA10G2	OP778255/-
16,342			*C. parvum*	IIaA18G3R1	OP778244/-
17,008	Otago skink	*Oligosoma otagense*	*C. hominis*	IbA10G2	OP778246/-
17,294			*C. hominis*	IgA17	OP778249/-
17,295			*C. hominis*	IgA17	OP778250/-
17,290			*C. parvum*	IIaA18G3R1	OP778247/-
17,292			*C. parvum*	IIaA17G3R1	OP778248/-
17,298	Grand skink	*Oligosoma grande*	*C. hominis*	IgA17	OP778251/-
16,911	Jewelled gecko	*Naultinus gemmeus*	-/*C. serpentis*	-	-/ OQ457495
16,912			*C. hominis*/*C. serpentis*	IbA10G2	OP778245/ OQ457496
19,249			*C. parvum*	IIaA17G4R1	OP778253/-
19,252			*C. parvum*	IIaA18G3R1	OP778252/-
19,250	Rough gecko	*Naultinus rudis*	*C. parvum*	IIaA18G3R1	OP778254/-

## Discussion

*Cryptosporidium* infections have been recorded in several reptile species (Carmel and Groves 1993; Graczyk et al. 1997; Jacobson 2007; Ladds 2009; Orós et al. 1998; Upton et al. 1989) but mammal-related *Cryptosporidium* species infecting reptiles are rare (Xiao et al. 2004). Our results indicate that mammal-related *Cryptosporidium* species, the main causative agents of disease in humans, non-human primates, and livestock in New Zealand (Garcia-R et al. 2017, 2020b) can infect captive reptiles. The clinical relevance of these findings is unknown.

New Zealand has a long history of isolation (Valente et al. 2019) and absence of native mammals (with exception of two bat species) may suggest that endemic reptiles have not been previously exposed to these pathogens. *Cryptosporidium* species adapted to mammals found infecting endemic reptiles may trigger clinical disease due to the recent coexistence and host-parasite relationship (Garcia-R et al. 2020a; Garcia-R and Hayman 2016). Importantly, these infections can act together with other stressors (e.g., habitat fragmentation or invasive species) and increase reptile mortality and extinction risk (Fey et al. 2015; Smith et al. 2009) of threatened taxa.

*Cryptosporidium parvum* and *C. hominis* have been previously reported in livestock and humans in New Zealand (Garcia-R et al. 2017). However, there is an increase in the detection of these pathogens in animal hosts worldwide. For instance, evidence of *C. hominis* in wildlife (kangaroos and other marsupials) and livestock (cattle and deer) residing in water catchments following its introduction by humans has been reported in Australia (Koehler et al. 2016; Ng et al. 2011; Zahedi et al. 2016a, 2016b, Zahedi et al. 2018). *Cryptosporidium hominis* is also widely recognised in equine populations in South America, Africa and Asia (Widmer et al. 2020). Likewise, *C. parvum* has been recently found in a wide variety of hosts including wildlife (Hailu et al. 2022; Karim et al. 2014; Ryan et al. 2021a).

To our knowledge there are no published cases of *Cryptosporidium* infections in endemic reptiles from New Zealand and this is the first report in captive endemic reptiles. We were careful to avoid cross-contamination and aimed to confirm all results by multiple methods. However, we were only able to amplify gp60 products from 12 animals and only two from 18S. We think that this may be due to the low concentration of oocyst/sporozoites and the possibility of primers amplifying numerous stretches of other organisms (including fungi or 16S rRNA bacteria) leading to reduced specificity (Xiao et al. 2000). Future studies should aim to confirm our findings and determine the source of infection and potential transmission pathways.

Reporting infections in endemic reptiles caused by *Cryptosporidium* has several implications for the health and conservation of wild native and endemic fauna of New Zealand. First, the reservoirs for *C. hominis* and *C. parvum* include widespread hosts, such as people and domestic animals, making the risk of infection more frequent through direct and indirect contact (Garcia-R et al. 2017, 2020b; Garcia-R and Hayman 2017). Second, *Cryptosporidium* oocysts are resilient and ubiquitous in the environment (Phiri et al. 2020) generating more opportunities for infections. And third, captive animals as part of breeding programmes must be carefully managed and screened before being released into areas free of the parasites. Our understanding of the *Cryptosporidium* species and subtypes infecting endemic New Zealand reptiles can help decision-making on conservation, testing protocols, and biosecurity during translocations of individuals to the wild. Hence, regular population and health monitoring of the captive and wild endemic reptiles will be important for timely management responses to threats such as gastrointestinal diseases.

## Supplementary Information

Below is the link to the electronic supplementary material.

Supplementary file1 (DOCX 16 KB)

Supplementary file2 (DOCX 20 KB)

## Data Availability

The nucleotide sequences of the partial gp60 and 18S genes were deposited in the GenBank database under accession numbers OP778244-OP778255 and OQ457495- OQ457496, respectively. The authors confirm that the ethical policies of the journal, as noted on the journal’s author guidelines page, have been adhered to. No ethics approval was needed.

## References

[CR1] Alley MR, Gartrell BD (2019). Wildlife diseases in New Zealand: recent findings and future challenges. N Z Vet J.

[CR2] Alves M, et al. (2005) Occurrence and molecular characterization of Cryptosporidium spp. in mammals and reptiles at the Lisbon Zoo. Parasitol Res 97(2):108–112. 10.1007/s00436-005-1384-9 10.1007/s00436-005-1384-9 15986253

[CR3] Brownstein DG, Strandberg JD, Montali RJ, Bush M, Fortner J (1977). Cryptosporidium in Snakes with Hypertrophic Gastritis. Vet Pathol.

[CR4] Carmel BP, Groves V (1993). Chronic cryptosporidiosis in Australian elapid snakes: control of an outbreak in a captive colony. Aust Vet J.

[CR5] Chapple DG, Ritchie PA, Daugherty CH (2009). Origin, diversification, and systematics of the New Zealand skink fauna (Reptilia: Scincidae). Mol Phylogen Evol.

[CR6] Doherty TS, Glen AS, Nimmo DG, Ritchie EG, Dickman CR (2016). Invasive predators and global biodiversity loss. Proc Natl Acad Sci USA.

[CR7] Fayer R, Morgan U, Upton SJ (2000). Epidemiology of *Cryptosporidium*: transmission, detection and identification. Int J Parasitol.

[CR8] Fayer R, Speer CA, Dubey JP, Fayer R (1997). The general biology of Cryptosporidium. Cryptosporidium and Cryptosporidiosis.

[CR9] Fayer R, Xiao L (2008) *Cryptosporidium* and Cryptosporidiosis, Second Edition. CRC Press

[CR10] Fey SB (2015). Recent shifts in the occurrence, cause, and magnitude of animal mass mortality events. Proc Natl Acad Sci USA.

[CR11] Garcia-R JC, Cox MP, Hayman DTS (2020). Comparative genetic diversity of *Cryptosporidium* species causing human infections. Parasitology.

[CR12] Garcia-R JC, French N, Pita A, Velathanthiri N, Shrestha R, Hayman D (2017) Local and global genetic diversity of protozoan parasites: Spatial distribution of *Cryptosporidium* and *Giardia* genotypes. PLOS Neglected Tropical Diseases 11(7):e0005736. 10.1371/journal.pntd.0005736 10.1371/journal.pntd.0005736 PMC5526614 28704362

[CR13] Garcia-R JC, Hayman DTS (2016). Origin of a major infectious disease in vertebrates: The timing of *Cryptosporidium* evolution and its hosts. Parasitology.

[CR14] Garcia-R JC, Hayman DTS (2017). Evolutionary processes in populations of *Cryptosporidium* inferred from gp60 sequence data. Parasitol Res.

[CR15] Garcia-R JC, Pita AB, Velathanthiri N, French NP, Hayman DTS (2020). Species and genotypes causing human cryptosporidiosis in New Zealand. Parasitol Res.

[CR16] Gartrell B, Chapple DG (2016). Diseases of New Zealand Reptiles. New Zealand Lizards.

[CR17] Gartrell BD, Hare KM (2005). Mycotic dermatitis with digital gangrene and osteomyelitis, and protozoal intestinal parasitism in Marlborough green geckos (Naultinus manukanus). N Z Vet J.

[CR18] Glaberman S, Moore JE, Lowery CJ, Chalmers RM, Sulaiman I, Elwin K (2002) Three drinking-water-associated cryptosporidiosis outbreaks, Northern Ireland. Emerg Infect Dis 8. 10.3201/eid0806.010368 10.3201/eid0806.010368 PMC2738494 12023922

[CR19] Graczyk TK, et al. (1997) Cryptosporidium sp. Infections in Green Turtles, Chelonia mydas, as a Potential Source of Marine Waterborne Oocysts in the Hawaiian Islands. Appl Environ Microbiol 63(7):2925–2927. 10.1128/aem.63.7.2925-2927.1997 10.1128/aem.63.7.2925-2927.1997 PMC1389213 16535658

[CR20] Hailu AW, et al. (2022) Genetic diversity of Cryptosporidium spp. in non-human primates in rural and urban areas of Ethiopia. PLOS ONE 17(4):e0267103. 10.1371/journal.pone.0267103 10.1371/journal.pone.0267103 PMC9009656 35421188

[CR21] Hay JM, Sarre SD, Lambert DM, Allendorf FW, Daugherty CH (2010). Genetic diversity and taxonomy: a reassessment of species designation in tuatara (Sphenodon: Reptilia). Conserv Genet.

[CR22] Hitchmough RA, Barr B, Knox C, Lettink M, Monks JM, Patterson GB, Reardon JT, van Winkel D, Rolfe J, Michel P (2021) Conservation status of New Zealand reptiles. New Zealand Threat Classification Series 35. Department of Conservation, Wellington, 15 p

[CR23] Hitchmough RA (2010). Conservation status of New Zealand reptiles. N Z J Zool.

[CR24] Jacobson E, Jacobson E (2007). Parasites and parasitic diseases of reptiles. Infectious diseases and pathology of reptiles.

[CR25] Ježková J, et al. (2016) Cryptosporidium testudinis sp. n., Cryptosporidium ducismarci Traversa, 2010 and Cryptosporidium tortoise genotype III (Apicomplexa: Cryptosporidiidae) in tortoises. Folia Parasitol 63(1):1–10. 10.14411/fp.2016.035 10.14411/fp.2016.035 27827334

[CR26] Johnson DW, Pieniazek NJ, Griffin DW, Misener L, Rose JB (1995). Development of a PCR protocol for sensitive detection of Cryptosporidium oocysts in water samples. Appl Environ Microbiol.

[CR27] Karim MR (2014). Multilocus typing of Cryptosporidium spp. and Giardia duodenalis from non-human primates in China. Int J Parasitol.

[CR28] Kearse M, Moir R, Wilson A, Stones-Havas S, Cheung M, Sturrock S (2012). Geneious Basic: an integrated and extendable desktop software platform for the organization and analysis of sequence data. Bioinformatics.

[CR29] Koehler AV, Haydon SR, Jex AR, Gasser RB (2016). Cryptosporidium and Giardia taxa in faecal samples from animals in catchments supplying the city of Melbourne with drinking water (2011 to 2015). Parasit Vectors.

[CR30] Koudela C, Modrý D (1998). New species of Cryptosporidium (Apicomplexa: Cryptosporidiidae) from lizards. Folia Parasitol.

[CR31] Kváč M, McEvoy J, Stenger B, Clark M, Cacciò SM, Widmer G (2014). Cryptosporidiosis in Other Vertebrates. Cryptosporidium: parasite and disease.

[CR32] Ladds PW (2009). Pathology of Australian native wildlife.

[CR33] Learmonth JJ, Ionas G, Ebbett KA, Kwan ES (2004). Genetic characterization and transmission cycles of *Cryptosporidium* species isolated from humans in New Zealand. Appl Environ Microbiol.

[CR34] Morgan U (1999). Phylogenetic Analysis of Cryptosporidium Isolates from Captive Reptiles Using 18S rDNA Sequence Data and Random Amplified Polymorphic DNA Analysis. J Parasitol.

[CR35] Ng J, Yang R, McCarthy S, Gordon C, Hijjawi N, Ryan U (2011). Molecular characterization of Cryptosporidium and Giardia in pre-weaned calves in Western Australia and New South Wales. Vet Parasitol.

[CR36] Nielsen SV, Bauer AM, Jackman TR, Hitchmough RA, Daugherty CH (2011). New Zealand geckos (Diplodactylidae): Cryptic diversity in a post-Gondwanan lineage with trans-Tasman affinities. Mol Phylogen Evol.

[CR37] O'Donoghue PJ (1995). Cryptosporidium and cryptosporidiosis in man and animals. Int J Parasitol.

[CR38] O’Neill SB, Chapple DG, Daugherty CH, Ritchie PA (2008). Phylogeography of two New Zealand lizards: McCann’s skink (Oligosoma maccanni) and the brown skink (O. zelandicum). Mol Phylogen Evol.

[CR39] Orós J, Rodríguez JL, Patterson-Kane J (1998). Gastric Cryptosporidiosis in a Wild Frilled Lizard From Australia. J Wildl Dis.

[CR40] Pavlasek I, Ryan U (2008) Cryptosporidium varanii takes precedence over C. saurophilum. Exp Parasitol 118(3):434–437. 10.1016/j.exppara.2007.09.006 10.1016/j.exppara.2007.09.006 17945215

[CR41] Pedraza-Díaz S, Ortega-Mora LM, Carrión BA, Navarro V, Gómez-Bautista M (2009). Molecular characterisation of Cryptosporidium isolates from pet reptiles. Vet Parasitol.

[CR42] Phiri B, et al. (2020) Does land use affect pathogen presence in New Zealand drinking water supplies? Water Res 185:116229. 10.1016/j.watres.2020.116229 10.1016/j.watres.2020.116229 32791457

[CR43] Richter B, Nedorost N, Maderner A, Weissenböck H (2011). Detection of Cryptosporidium species in feces or gastric contents from snakes and lizards as determined by polymerase chain reaction analysis and partial sequencing of the 18S ribosomal RNA gene. J Vet Diagn Invest.

[CR44] Rinaldi L, Capasso M, Mihalca AD, Cirillo R, Cringoli G, Cacciò S (2012). Prevalence and molecular identification of Cryptosporidium isolates from pet lizards and snakes in Italy. Parasite (paris, France).

[CR45] Ryan U, Zahedi A, Feng Y, Xiao L (2021a) An Update on Zoonotic Cryptosporidium Species and Genotypes in Humans. Animals 11(11). 10.3390/ani11113307 10.3390/ani11113307 PMC8614385 34828043

[CR46] Ryan UM, Feng Y, Fayer R, Xiao L (2021). Taxonomy and molecular epidemiology of Cryptosporidium and Giardia – a 50 year perspective (1971–2021). Int J Parasitol.

[CR47] Scullion FT, Scullion MG (2009). Gastrointestinal Protozoal Diseases in Reptiles. Journal of Exotic Pet Medicine.

[CR48] Smith KF, Acevedo-Whitehouse K, Pedersen AB (2009). The role of infectious diseases in biological conservation. Anim Conserv.

[CR49] Terrell S, Uhl E, Funk R (2003) Proliferative enteritis in leopard geckos (Eublepharis macularius) associated with Cryptosporidium sp. infection. J Zoo Wildl Med 34(1):69–75. 10.1638/1042-7260(2003)34[0069:PEILGE]2.0.CO;2 10.1638/1042-7260(2003)34[0069:PEILGE]2.0.CO;2 12723803

[CR50] Tingley R, Hitchmough RA, Chapple DG (2013). Life-history traits and extrinsic threats determine extinction risk in New Zealand lizards. Biol Conserv.

[CR51] Towns DR, Daugherty CH, Cree A (2001). Raising the prospects for a forgotten fauna: a review of 10 years of conservation effort for New Zealand reptiles. Biol Conserv.

[CR52] Traversa D (2010). Evidence for a new species of Cryptosporidium infecting tortoises: Cryptosporidium ducismarci. Parasit Vectors.

[CR53] Traversa D, Iorio R, Otranto D, Modrý D, Šlapeta J (2008). Cryptosporidium from tortoises: Genetic characterisation, phylogeny and zoonotic implications. Mol Cell Probes.

[CR54] Upton SJ, McAllister CT, Freed PS, Barnard SM (1989) Cryptosporidium spp. in wild and captive reptiles. J Wildl Dis 25(1):20–30. 10.7589/0090-3558-25.1.20 10.7589/0090-3558-25.1.20 2915400

[CR55] Valente L, Etienne RS, Garcia-R JC (2019). Deep macroevolutionary impact of humans on New Zealand’s unique Avifauna. Curr Biol.

[CR56] Waldron LS, Ferrari BC, Power ML (2009) Glycoprotein 60 diversity in *C. hominis* and *C. parvum* causing human cryptosporidiosis in NSW, Australia. Exp Parasitol 122(2):124–127. 10.1016/j.exppara.2009.02.006 10.1016/j.exppara.2009.02.006 19233175

[CR57] Widmer G, Köster PC, Carmena D (2020). Cryptosporidium hominis infections in non-human animal species: revisiting the concept of host specificity. Int J Parasitol.

[CR58] Xiao L, Alderisio K, Limor J, Royer M, Lal AA (2000). Identification of Species and Sources of *Cryptosporidium* Oocysts in Storm Waters with a Small-Subunit rRNA-Based Diagnostic and Genotyping Tool. Appl Environ Microbiol.

[CR59] Xiao L (1999). Phylogenetic analysis of *Cryptosporidium* parasites based on the small-subunit rRNA gene locus. Appl Environ Microbiol.

[CR60] Xiao L (2004). Genetic Diversity of * Cryptosporidium* spp. Captive Reptiles Appl Environ Microbiol.

[CR61] Zahedi A, et al. (2016a) Zoonotic *Cryptosporidium s*pecies in animals inhabiting Sydney water catchments. PLOS ONE 11(12):e0168169. 10.1371/journal.pone.0168169 10.1371/journal.pone.0168169 PMC5156390 27973572

[CR62] Zahedi A (2018). *Cryptosporidium* species and subtypes in animals inhabiting drinking water catchments in three states across Australia. Water Res.

[CR63] Zahedi A, Paparini A, Jian F, Robertson I, Ryan U (2016). Public health significance of zoonotic Cryptosporidium species in wildlife: Critical insights into better drinking water management. International Journal for Parasitology: Parasites and Wildlife.

