# Supplementary material for: Mammal-related Cryptosporidium infections in endemic reptiles of New Zealand
Source: Parasitol Res. 2023 Mar 24;122(5):1239–44. doi: 10.1007/s00436-023-07824-4 (PMC10097775; doi:10.1007/s00436-023-07824-4)
Supplement: Supplementary file 1 — Supplementary file1 (DOCX 16 KB) [file 436_2023_7824_MOESM1_ESM.docx]

Table S1. List of samples analysed in this study.

| LabID | Species | Sample Type(s) | Supplier |
| --- | --- | --- | --- |
| 16342 | Tuatara | Faeces | Wild base Massey University |
| 16386 | Tuatara | Faeces | Wild base Massey University |
| 18421 | Tuatara | Faeces | Invercargill City Council |
| 16911 | Jewelled Gecko | Gastric wash and Intestinal tissue | Auckland Zoo |
| 16912 | Jewelled Gecko | Gastric wash and Intestinal tissue | Auckland Zoo |
| 19249 | Jewelled Gecko | Cloacal wash | Auckland Zoo |
| 19252 | Jewelled Gecko | Gastric wash | Auckland Zoo |
| 17006 | Otago skink | Cloacal wash and Gastric wash | Auckland Zoo |
| 17008 | Otago skink | Cloacal wash and Gastric wash | Auckland Zoo |
| 17290 | Otago skink | Gastric wash | Wellington Zoo |
| 17291 | Otago skink | Cloacal wash and Gastric wash | Wellington Zoo |
| 17292 | Otago skink | Gastric wash and Cloacal wash | Auckland Zoo |
| 17293 | Otago skink | Gastric wash and Cloacal wash | Auckland Zoo |
| 17294 | Otago skink | Gastric wash and Cloacal wash | Auckland Zoo |
| 17295 | Otago skink | Gastric wash and Cloacal wash | Auckland Zoo |
| 19470 | Otago skink | Cloacal wash | Auckland Zoo |
| 17296 | Grand Skink | Cloacal wash | Auckland Zoo |
| 17297 | Grand Skink | Cloacal wash | Auckland Zoo |
| 17298 | Grand Skink | Gastric wash and Cloacal wash | Auckland Zoo |
| 17299 | Grand Skink | Cloacal wash | Auckland Zoo |
| 17300 | Grand Skink | Gastric wash and Cloacal wash | Auckland Zoo |
| 19250 | Rough Gecko | Gastric wash | Auckland Zoo |
