# Supplementary material for: Mammal-related Cryptosporidium infections in endemic reptiles of New Zealand
Source: Parasitol Res. 2023 Mar 24;122(5):1239–44. doi: 10.1007/s00436-023-07824-4 (PMC10097775; doi:10.1007/s00436-023-07824-4)
Supplement: Supplementary file 2 — Supplementary file2 (DOCX 20 KB) [file 436_2023_7824_MOESM2_ESM.docx]

Table S2. Primers for PCR and DNA sequencing employed in this study.

| **Locus** | **Position** | **Primer name** | **Sequence 5’–3’** | **Reference** |
| --- | --- | --- | --- | --- |
| gp60 | External | AL3531F | ATAGTCTCCGCTGTATTC | Glaberman et al 2002 |
|  | External | AL3534R | GCAGAGGAACCAGCATC |  |
|  | Internal | AL3532F | TCCGCTGTATTCTCAGCC |  |
|  | Internal | AL3535R | GGAAGGAACGATGTATCT |  |
|  | External | S60.F728 | ACCACATTTTACCCACACATC | Waldron et al 2009 |
|  | External | S60.OutR | TCCTCACTCGATCTAGCTCA |  |
|  | Internal | S60.ATGF | ATGAGATTGTCGCTCATTATCG |  |
|  | Internal | S60.StopR | TTACAACACGAATAAGGCTGC |  |
| 18S | External | L1 | GTTAAACTGCGAATGGCTCA | Learmonth et al 2004 |
|  | External | L2 | CCATTTCCTTCGAAACAGGA |  |
|  | Internal | S1 | CTCGACTTTATGGAAGGGTTG |  |
|  | Internal | S2 | CCTCCAATCTCTAGTTGGCATA |  |
|  | External | F1 | TTCTAGAGCTAATACATGCG | Xiao et al 1999, 2000 |
|  | External | R1 | CCCATTTCCTTCGAAACAGGA |  |
|  | Internal | F2 | GGAAGGGTTGTATTTATTAGATAAAG |  |
|  | Internal | R2 | AAGGAGTAAGGAACAACCTCCA |  |
|  |  | CPB-DIAGF | AAGCTCGTAGTTGGATTTCTG | Johnson et al 1995 |
|  |  | CPB-DIAGR | TAAGGTGCTGAAGGAGTAAGG |  |

PCR conditions for gp60 amplification:

Glaberman et al 2002

1. 95°C x 3 mins
2. 94°C x 45 sec
3. 50°C x 45sec
4. 72°C x 60 sec
5. 72°C x 10 mins

From 2) to 4) 35 cycles

Waldron et al 2009

1. 94°C x 3 mins
2. 94°C x 45 sec
3. 58°C x 45sec
4. 72°C x 90 sec
5. 72°C x 5 mins

From 2) to 4) 35 cycles

PCR conditions for 18S amplification:

Learmonth et al 2004

External:

1. 96°C x 2 mins
2. 94°C x 20 sec
3. 57°C x 20sec
4. 72°C x 30 sec

From 2) to 4) 40 cycles

Internal:

1. 96°C x 2 mins
2. 94°C x 20 sec
3. 60°C x 20sec
4. 72°C x 30 sec

From 2) to 4) 40 cycles

Xiao et al 1999, 2000

1. 94°C x 3 mins
2. 94°C x 45 sec
3. 55°C x 45sec
4. 72°C x 60 sec
5. 72°C x 7 mins

From 2) to 4) 35 cycles

Johnson et al 1995

1. 80°C x 5 mins
2. 98°C x 30 sec
3. 55°C x 30sec
4. 72°C x 60 sec
5. 72°C x 10 mins

From 2) to 4) 39 cycles

References

Glaberman S, Moore JE, Lowery CJ, Chalmers RM, Sulaiman I, Elwin K (2002) Three drinking-water-associated cryptosporidiosis outbreaks, Northern Ireland. Emerg Infect Dis 8 doi:10.3201/eid0806.010368

Johnson DW, Pieniazek NJ, Griffin DW, Misener L, Rose JB (1995) Development of a PCR protocol for sensitive detection of Cryptosporidium oocysts in water samples. Appl Environ Microbiol 61(11):3849-55

Learmonth JJ, Ionas G, Ebbett KA, Kwan ES (2004) Genetic characterization and transmission cycles of *Cryptosporidium* species isolated from humans in New Zealand. Appl Environ Microbiol 70(7):3973-3978 doi:10.1128/aem.70.7.3973-3978.2004

Waldron LS, Ferrari BC, Power ML (2009) Glycoprotein 60 diversity in *C. hominis* and *C. parvum* causing human cryptosporidiosis in NSW, Australia. Exp Parasitol 122(2):124-127

Xiao L, Alderisio K, Limor J, Royer M, Lal AA (2000) Identification of Species and Sources of *Cryptosporidium* Oocysts in Storm Waters with a Small-Subunit rRNA-Based Diagnostic and Genotyping Tool. Appl Environ Microbiol 66(12):5492 doi:10.1128/AEM.66.12.5492-5498.2000

Xiao L, et al. (1999) Phylogenetic analysis of *Cryptosporidium* parasites based on the small-subunit rRNA gene locus. Appl Environ Microbiol 65(4):1578-1583
